# Association of serum xanthine oxidase levels with hypertension: a study on Bangladeshi adults

**DOI:** 10.1038/s41598-022-26341-5

**Published:** 2022-12-16

**Authors:** Rakib Miah, Khandaker Atkia Fariha, Sabrina Amita Sony, Shamim Ahmed, Mahmudul Hasan, Ananya Dutta Mou, Zitu Barman, Akibul Hasan, Nayan Chandra Mohanto, Nurshad Ali

**Affiliations:** Department of Biochemistry and Molecular Biology, Shahjalal University of Science and Technology, Sylhet, 3114 Bangladesh

**Keywords:** Biomarkers, Predictive markers

## Abstract

Xanthine oxidase (XO) is a metalloflavoenzyme associated with the uric acid formation in purine metabolism. Serum XO has been suggested to be associated with liver and kidney dysfunction, diabetes and cardiovascular diseases. However, there is limited information on the relationship between serum XO levels and hypertension. This study aimed to assess the relationship between serum XO levels and hypertension in Bangladeshi adults. In this study, fasting blood samples were collected from 312 participants (225 males and 87 females), aged ≥ 20 years. Serum levels of XO were determined by ELISA and other biochemical parameters including serum uric acid (SUA) were measured by colorimetric methods. Hypertension was defined as SBP ≥ 140 mmHg and/or DBP ≥ 90 mmHg or self-reported recent use of anti-hypertensive medications. Association between serum XO levels and hypertension was evaluated by multinomial logistic regression analysis. The mean level of XO was significantly higher (p < 0.001) in females (5.8 ± 3.2 U/L) than in males (3.9 ± 2.5 U/L). When the participants were divided by blood pressure levels, the mean level of serum XO was significantly higher (p < 0.01) in the hypertensive group (5.0 ± 2.7 U/L) compared to the normotensive control group (4.0 ± 2.7 U/L). An increasing trend for SBP and DBP levels was observed across the XO quartiles (at least p < 0.01 for both cases). A significant positive correlation was found for XO with SBP and DBP (p < 0.01). In regression analysis, the serum levels of XO showed a significant and independent association with hypertension prevalence. In conclusion, the mean level of serum XO was significantly higher in hypertensive individuals and XO was independently associated with the prevalence of hypertension. Our results indicate that XO may have a potential role in the pathophysiology of elevated blood pressure through generating of reactive oxygen species. Further large-scale longitudinal studies are needed to determine the underlying mechanisms between XO and hypertension.

## Introduction

Xanthine oxidase (XO) is an isoform of xanthine oxidoreductase (XOR) that catalyzes the oxidation of hypoxanthine to xanthine and xanthine to uric acid, the last two steps of purine metabolism in humans^1^. Another isoform of XOR is xanthine dehydrogenase (XDH). XDH is primarily synthesized as the 150-kDa protein from which XO is derived, either reversibly by limited proteolysis or reversibly by conformational changes^2^. XO is widely distributed throughout the different organs in mammals including the heart, liver, lung, kidney, brain, and gut as well as the plasma^3^. Apart from its role in uric acid production, XO is also involved in the formation of reactive oxygen species (ROS) in mammalian cells^1,4^. ROS are associated with increased blood pressure (BP) via endothelial dysfunction, vascular inflammation, and structural remodeling^5^. Nitric oxide (NO) is a potent vasodilator and its levels may be diminished upon reaction with ROS-like superoxide anion radical (O_2_^•−^) causing greater resistance of arterioles and finally leading to hypertension development^6,7^. Although there might be other sources of oxidants in the vasculature implicated in hypertension, XO has received greater attention in recent years. In animal studies, it has been shown that XO-derived oxidants exert a stronger effect on arterial blood pressure in spontaneously hypertensive rats^8^. XO has also been suggested to be associated with diabetes in the human population^9,10^. However, only a limited number of studies have assessed the relationship between circulating levels of XO and hypertension in human individuals.

The elevated level of serum uric acid (SUA) is associated with several disease conditions including obesity, hypertension, diabetes, liver and kidney dysfunction, metabolic syndrome, and cardiovascular diseases^11–17^. However, some studies also reported that both elevated and low levels of SUA are associated with cardiovascular disease^18,19^ and renal disease^20,21^. A recent umbrella review that included meta-analyses, Mendelian analyses and randomized controlled trials, indicated that SUA has a clear role only in gout development and nephrolithiasis^22^. Therefore, SUA has been suggested as a mere biomarker other than an actual risk factor for kidney and cardiovascular disease including elevated blood pressure^22,23^.

A recent publication showed a positive association between plasma XOR and elevated blood pressure (BP) in the Japanese population who were not using anti-hypertensive or anti-hyperuricemic medications^24^. The authors suggested that XOR may induce the pathophysiology of elevated BP through ROS other than uric acid production^24^. Another study conducted on Japanese adults in the same country reported an independent association of plasma XOR with the risk of hypertension in nondiabetic subjects who were not taking any medications^25^. An observational study in a patient cohort in Japan showed that the use of XO inhibitors was associated with a lower risk of cardiovascular morbidity and all-cause mortality in hypertensive individuals with reduced kidney function^26^. Furthermore, Feig and colleagues found that allopurinol, a potential inhibitor of XO decreased the blood pressure in adolescents with newly diagnosed hypertension^27^. Therefore, it remains unclear, whether, XO causes hypertension before its involvement in renal injury, diabetes, and cardiovascular disease. Moreover, several lines of evidence suggest that either uric acid or XO are associated with vascular injury through endocytosis by vascular endothelial cells^28–30^. Therefore, further studies are needed to examine the role of XO in hypertension development. Moreover, it is also important to examine the association between XO and hypertension in various age groups as well as in different ethnic populations. Considering these aspects, our present study aimed to investigate the relationship between XO and hypertension in an adult cohort in Bangladesh.

## Methods

### Study population

This cross-sectional study was conducted between October 2019 and July 2020 at the Department of Biochemistry and Molecular Biology, Shahjalal University of Science and Technology, Sylhet, Bangladesh. In total, 312 participants (age range 20–80 years) were enrolled from general people living in the Sylhet city region, university students and academic and non-academic staff. As inclusion criteria, both genders aged ≥ 20 years and willing to participate were included in the study. As exclusion criteria, participants who were in the pregnancy or lactating stage or individuals with a history of drug addiction, alcohol consumption or participants with anti-hyperuricemic drug intake were not included in the study. We further excluded subjects with self-reported liver and kidney diseases, hypothyroidism, malignant disease and any infectious diseases. The study protocol was approved by the internal ethics committee existed at the Department of Biochemistry and Molecular Biology, SUST (Reference no 02/BMB/2019). All methods of the study were carried out in accordance with relevant guidelines and regulations. Informed consent was obtained from all subjects before inclusion in the study.

### Data collection

The participant’s anthropometric data like weight, height, waist circumferences (WC) and hip circumferences (HC) were measured according to standard procedure described elsewhere^31–37^. Individual health status-related information such as the presence of hypertension, diabetes mellitus, and other chronic diseases was also recorded in the questionnaire form. Body mass index (BMI) was calculated as body weight in kg divided by body height in meters squared (kg/m^2^). The systolic and diastolic blood pressure (SBP and DBP, respectively) were measured using digital blood pressure (BP) machine (Omron M10, Tokyo Japan).

### Blood collection and laboratory measurements

Fasting blood samples (4 mL) were drawn from the antecubital vein by venipuncture after overnight fasting (10–12 h) and kept in the serum collection tube. The samples were collected from each participant in the morning by trained personnel. The blood samples were placed in the ice box immediately after collection and transported to the laboratory for serum separation. The blood samples were centrifuged by an ultracentrifuge machine (Sorvall ST 8R Centrifuge, Thermo Scientific, Germany) at 4400 rpm for 10 min. The isolated serum samples were collected and stored at − 80 °C at the Departmental laboratory until biochemical parameter measurements. The serum level of xanthine oxidase (XO) was measured by enzyme-linked immunosorbent assay (ELISA). The other biochemical parameters like fasting blood glucose (FBG), serum uric acid (SUA), serum creatinine, and lipid profile markers (TC: total cholesterol, TG: triglyceride, HDL-C: high-density lipoprotein cholesterol, and LDL-C: low-density lipoprotein cholesterol) were measured by colorimetric methods using commercially available kits (Human Diagnostic, Germany) with a biochemistry analyzer (Humalyzer 3000, USA). All the measurements were done according to the manufacturer’s protocol provided in the kits.

### Estimation of serum xanthine oxidase (XO)

Xanthine Oxidase (XO) levels in serum were measured by ELISA using a commercially available ELISA kit, which was purchased from MyBioSource company, USA (Human XO ELISA Kit, Cat No: MBS774009). This kit was used to measure the levels of XO in serum, based on the principle of ELISA. Briefly, standards and serum samples were added to the wells that were pre-coated with specific antibodies and then HRP-Conjugate solution was added to the wells to form an immune complex. After incubation, the unbound enzyme was removed through washing. The substrates were added, then the solution colour turned blue, and finally changed into yellow. The colour intensity was positively correlated with XO levels present in the serum sample. The absorbance (OD) of each micro-well was measured at 450 nm within 15 min by an ELISA reader (Apollo 11 LB 913, Berthold, Germany). Finally, XO concentrations in the samples were calculated according to the standard graph prepared in the excel sheet.

### Diagnostic criteria

Hypertension was defined as SBP ≥ 140 mmHg and/or DBP ≥ 90 mmHg^38^, and participants with self-reporting intake of antihypertensive medication. Prehypertension was defined as SBP 120–139 mmHg; and/or DBP 80–89 mmHg^38^. Hyperuricemia was defined as SUA concentration > 7.0 mg/dL in males and > 6.0 mg/dL in females^11,12^.

### Statistical analysis

All statistical analyses were performed using IBM SPSS statistical software version 25.0 (SPSS Inc, Chicago, Illinois, USA). Descriptive data are presented as mean ± standard deviation (SD), whereas categorical variables are expressed as percentages. Differences in the baseline variables between the control and the hypertensive group were analyzed by independent sample t-test. Serum XO was divided into four quartiles based on the frequency test: Q1 (< 2.32 U/L), Q2 (2.32–3.76 U/L), Q3 (3.77–5.79 U/L), and Q4 (> 5.79 U/L). One-way ANOVA was used to determine the differences in variables in the XO quartiles and BP groups. The association between serum level of XO and hypertension was evaluated by multinomial logistic regression analysis. p-values were considered significant at p < 0.05.

## Results

### Baseline characteristics of the study subjects in the control and hypertensive groups

The basic characteristics of the participants by blood pressure groups are summarized in Table 1. Of the total 312 subjects, 181 were normotensive control and 131 were hypertensive individuals. The mean age of the participants was 43.3 ± 12.6 years with a significant difference between the normotensive (40.4 ± 12.3 years) and hypertensive (47.2 ± 12.0 years) groups (p < 0.001). The mean value of BMI was significantly higher in the hypertensive group (26.0 ± 3.7 kg/m^2^) compared to the normotensive (24.6 ± 3.7 kg/m^2^) group (p < 0.01). Subjects in the hypertensive group had a higher mean level of XO (5.0 ± 2.7 U/L) and SUA (8.1 ± 4.2 mg/dL) than subjects in the control group (4.0 ± 2.7 U/L, 5.2 ± 1.6 mg/dL, respectively) (p < 0.01). The mean concentration of FBG, TC, and HDL-C were also significantly higher in the hypertensive group compared to the control group (at least p < 0.05 for all cases).

**Table 1:** Baseline characteristics of the participants in normotensive and hypertensive groups.

Variables	Total (n = 312)	Normotensive (n = 181)	Hypertensive (n = 131)	p-value
	Mean ± SD	Mean ± SD	Mean ± SD	
Gender, m/f	225/87	130/51	95/36	−
Age, year	43.3 ± 12.6	40.4 ± 12.3	47.2 ± 12.0	0.000
Height, cm	162.1 ± 9.3	161.4 ± 9.3	160.8 ± 9.3	0.563
Weight, kg	65.2 ± 11.7	64 ± 10.3	66.9 ± 13.3	0.030
BMI, kg/m^2^	25.2 ± 3.7	24.6 ± 3.7	26.0 ± 3.7	0.001
WC, cm	86.6 ± 10.4	84.8 ± 10 .0	88.9 ± 10.4	0.004
HC, cm	92.7 ± 8.7	90.9 ± 7.7	92.7 ± 8.7	0.110
SBP, mmHg	130.2 ± 17.9	119.5 ± 8.9	145 ± 16.5	0.000
DBP, mmHg	84.5 ± 10 .0	79.6 ± 7.2	91.3 ± 9.4	0.000
FBG, mmol/dL	7.3 ± 3.7	6.6 ± 3.2	8.0 ± 4.2	0.001
SUA, mg/dL	5.4 ± 1.8	5.2 ± 1.6	8.1 ± 4.2	0.004
XO, U/L	4.4 ± 2.8	4.0 ± 2.7	5.0 ± 2.7	0.002
Creatinine, mg/dL	0.9 ± 0.3	0.9 ± 0.3	0.8 ± 0.3	0.490
TG, mg/dL	199.7 ± 122.8	191 ± 112.4	210.8 ± 122.8	0.184
TC, mg/dL	217.7 ± 87.1	207.7 ± 80.7	230.6 ± 93.5	0.032
HDL-C, mg/dL	34.3 ± 13.6	32.1 ± 11.7	37.2 ± 15.0	0.000
LDL-C, mg/dL	144.2 ± 80.3	138.1 ± 74.9	152.1 ± 86.4	0.155
**Smoking, n (%)**				0.214
Yes	63 (20.3)	48 (22.1)	15 (15.9)	–
No	248 (79.7)	169 (77.9)	79 (84.1)	–

Values are presented as mean ± SD. p-values are obtained from the independent sample t-test.

BMI, body mass index; WC, waist circumference; HC, hip circumference; SBP, systolic blood pressure; DBP, diastolic blood pressure; PP, pulse pressure; XO, xanthine oxidase; SUA, serum uric acid; FBG, fasting blood glucose; TG, triglyceride; TC, total cholesterol; HDL-C, high-density lipoprotein cholesterol; LDL-C, low-density lipoprotein cholesterol.

### Baseline characteristics of the participants based on sex

**Figure 1:**
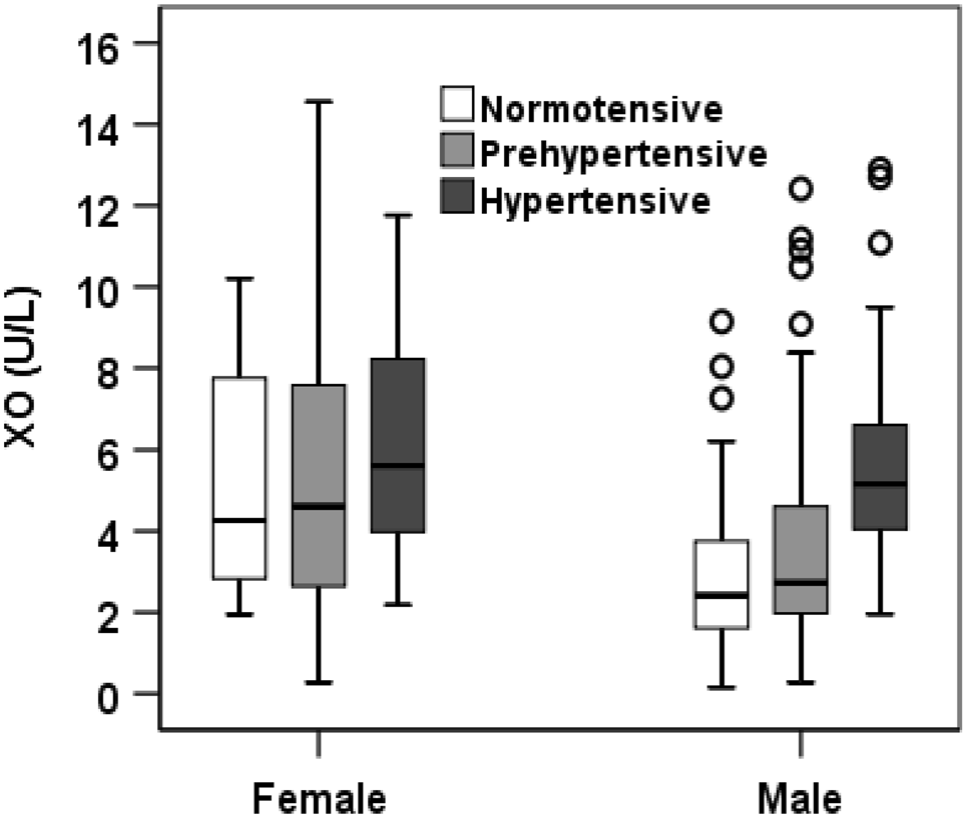
Levels of XO in the normotensive, prehypertensive, and hypertensive groups by gender. p < 0.01, when XO levels in normotensive, prehypertensive, and hypertensive groups were compared between male and female groups. p < 0.05, when XO levels were compared within the blood pressure groups in female subjects.

**Figure 2:**
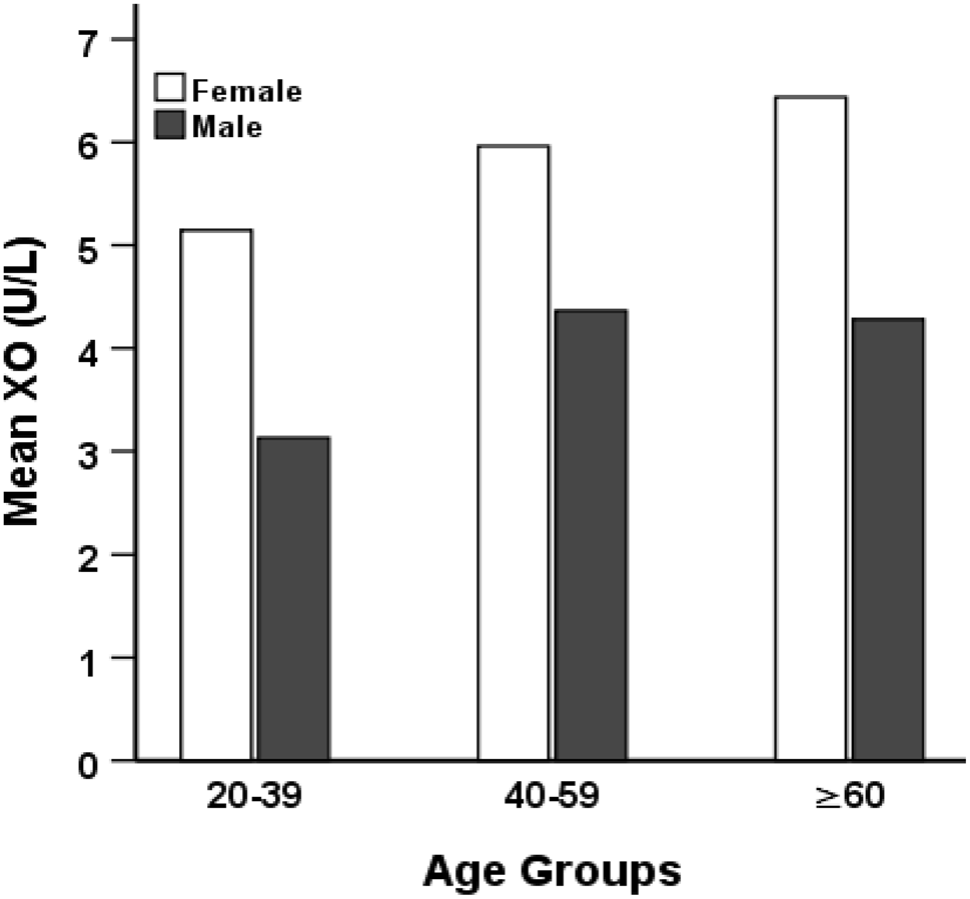
Levels of XO by age groups. p < 0.01, when XO level was compared within age groups in females. p-values were obtained from one-way ANOVA.

Table 2 shows the baseline characteristics according to sex. Among the study subjects, 225 (72.1%) were males and 87 (27.9%) were females. There was no significant difference in the mean age and BMI. The mean level of XO was higher in females (5.8 ± 3.2 U/L) than in male (3.9 ± 2.5 U/L) participants (p < 0.001), whereas, the mean SUA was higher in males (5.9 ± 1.6 mg/dL) than in female (4.4 ± 1.7 mg/dL) subjects (p < 0.001). The mean level of FBG was higher in females than in males (p < 0.05). Based on BP levels the participants were also divided into normotensive, prehypertensive and hypertensive groups (Fig. 1). The mean serum XO level was higher in females than in males (p < 0.01). No significant difference was found within the blood pressure groups in female subjects. However, the XO level was higher in the hypertensive group than in the normotensive and prehypertensive groups, especially in male subjects (p < 0.01). When participants were divided into three age groups (Fig. 2), an increasing trend in XO level was observed in the age groups, especially in females (p < 0.01).

**Table 2:** Baseline characteristics of the participants based on gender.

Variables	Total (n = 312)	Male (n = 225)	Female (n = 87)	p-value
Age, year	43.3 ± 12.6	42.9 ± 12.6	44.3 ± 12.6	0.387
Height, cm	162.1 ± 9.3	164.8 ± 7.1	151.7 ± 7.5	0.000
Weight, kg	65.2 ± 11.7	68.1 ± 10.6	57.9 ± 11.4	0.000
BMI, kg/m^2^	25.2 ± 3.7	25 ± 3.4	25.6 ± 4.5	0.249
WC, cm	86.6 ± 10.4	87.8 ± 10.2	84 ± 10.6	0.015
HC, cm	92.7 ± 8.7	92.2 ± 8.2	90.7 ± 8.2	0.203
SBP, mmHg	130.2 ± 17.9	130.1 ± 17.5	130.6 ± 18.8	0.830
DBP, mmHg	84.5 ± 10	84.3 ± 10.3	85 ± 9.4	0.559
FBG, mmol/dL	7.3 ± 3.7	6.9 ± 3.6	8.2 ± 4	0.024
SUA, mg/dL	5.4 ± 1.8	5.9 ± 1.6	4.4 ± 1.7	0.000
XO, U/L	4.4 ± 2.8	3.9 ± 2.5	5.8 ± 3.2	0.000
Creatinine, mg/dL	0.9 ± 0.3	0.9 ± 0.3	0.7 ± 0.2	0.000
TG, mg/dL	199.7 ± 122.8	201.6 ± 18.3	194.9 ± 134.4	0.703
TC, mg/dL	217.7 ± 87.1	213.6 ± 82.5	228.1 ± 97.9	0.248
HDL-C, mg/dL	34.3 ± 13.6	34.1 ± 14.4	36.4 ± 10.9	0.160
LDL-C, mg/dL	144.2 ± 80.3	140.1 ± 72.6	154.8 ± 97.4	0.170
Hypertension, (%)	41.9	42.2	41.4	–

Values are presented as mean ± SD. p-values are obtained from the independent sample t-test.

BMI, body mass index; WC, waist circumference; HC, hip circumference; SBP, systolic blood pressure; DBP, diastolic blood pressure; PP, pulse pressure; XO, xanthine oxidase; SUA, serum uric acid; FBG, fasting blood glucose; TG, triglyceride; TC, total cholesterol; HDL-C, high-density lipoprotein cholesterol; LDL-C, low-density lipoprotein cholesterol.

### Baseline parameters in XO quartiles and correlation of XO with BP

**Figure 3:**
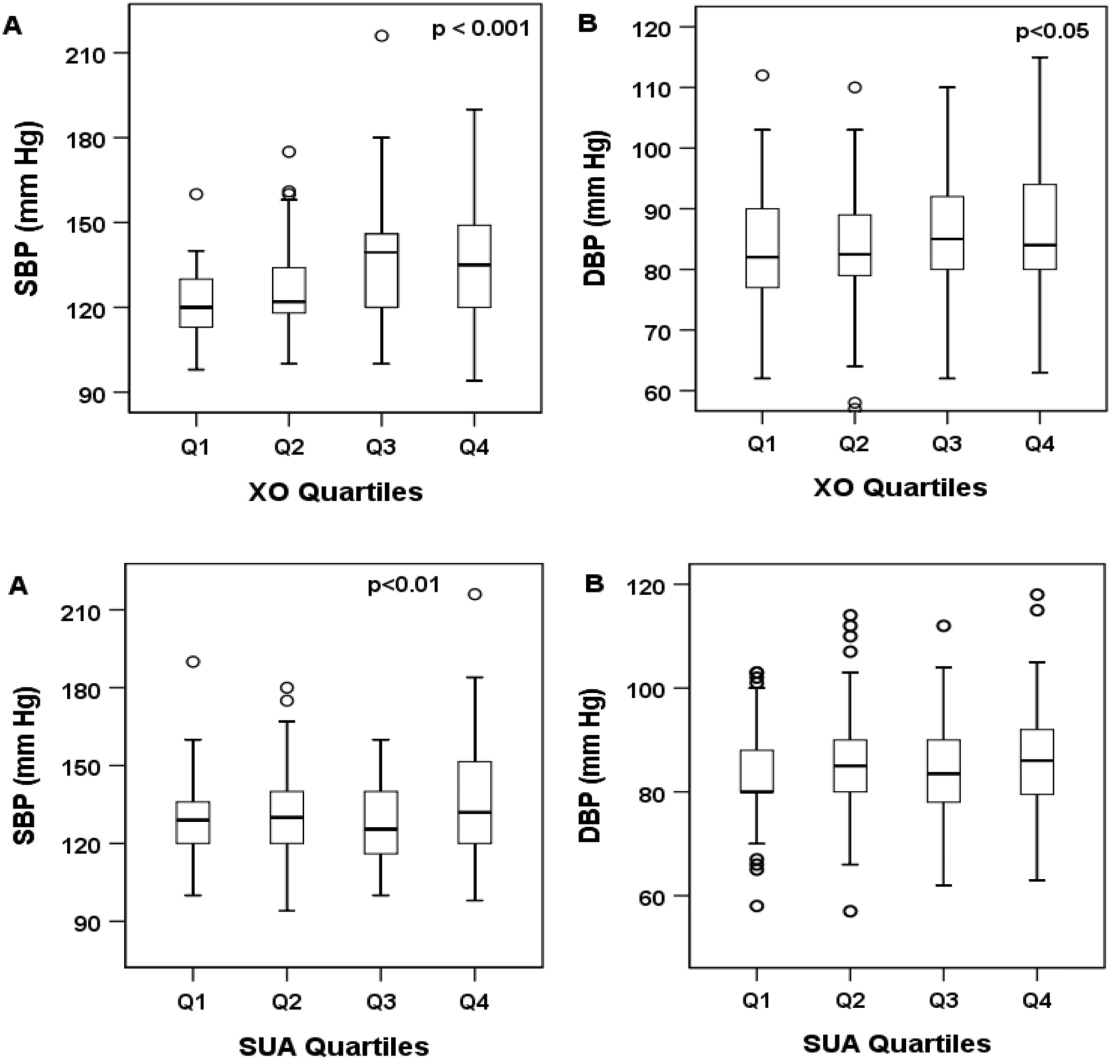
Systolic (**A**) and Diastolic (**B**) blood pressure across the XO and SUA quartiles. The scale in the Y-axis is not similar to the figures. p-values were obtained from one-way ANOVA.

**Figure 4:**
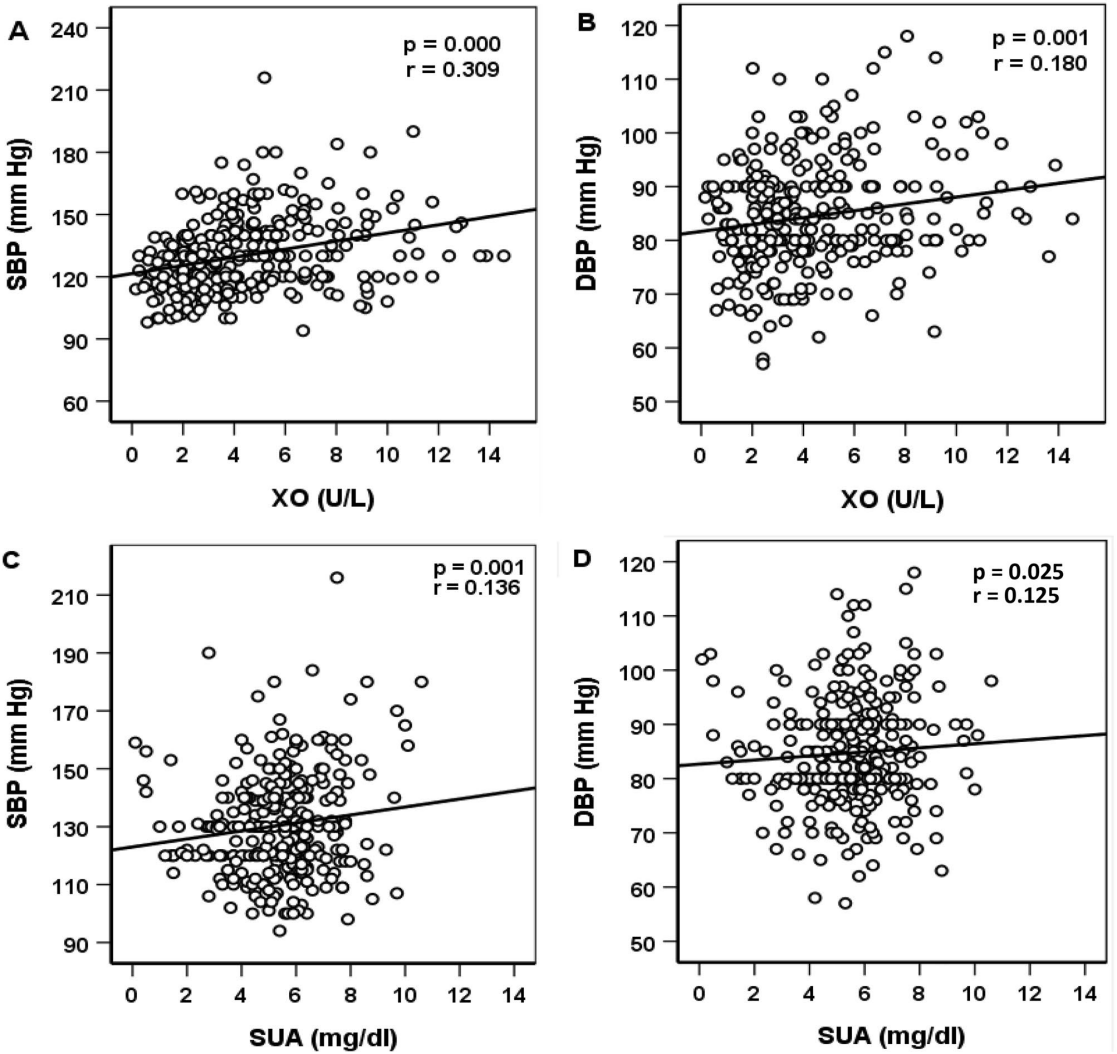
Correlation of XO level with SBP (**A**) and DBP (**B**) and SUA levels with SBP (**C**) and DBP (D). The scale in the X-axis is not similar in (**A**) and (**C**). p-values for XO and SUA with blood pressures were obtained from Pearson’s correlation coefficient.

Serum XO levels were divided into four quartiles: Q1 (< 2.32 U/L), Q2 (2.32–3.76 U/L), Q3 (3.77–5.79 U/L), and Q4 (> 5.79 U/L). A significant increasing trend was observed for SBP and DBP across the XO quartiles (Table 3 and Fig. 3). In SUA quartile groups, the increasing trend of both SBP and DBP was inconsistent, however, the overall trend within the quartiles was significant, especially for SBP. The correlation of XO and SUA with SBP and DBP is depicted in Fig. 4. A significant positive correlation was observed for serum XO level with SBP and DBP (at least p < 0.01 for both cases). Similarly, SUA also showed a positive correlation with SBP and DBP (at least p < 0.05 for both cases).

**Table 3:** Characteristics of the study subjects according to XO quartiles.

Variables	Xanthine oxidase (U/L)				p-value
	Q1 (< 2.32)	Q2 (2.32–3.76)	Q3 (3.77–5.79)	Q4 (> 5.79)	
N	78	78	78	78	–
Age (year)	38.2 ± 11.3 (65)	42.2 ± 12.9 (70)	46.6 ± 12.8 (85)	43.3 ± 12.6 (85)	0.000
Height (cm)	164.2 ± 9.9 (181)	161.4 ± 8.6 (178)	162.3 ± 7.6 (177)	161.1 ± 9.3 (181)	0.000
Weight (kg)	67.6 ± 8.9 (92)	66.4 ± 11.1 (96.6)	64.9 ± 13.4 (95)	65.2 ± 11.7 (105)	0.019
BMI (kg/m^2^)	25.2 ± 3.4 (38.4)	25.4 ± 3.6 (33.9)	24.9 ± 3.8 (40)	25.2 ± 3.8 (40)	0.862
WC (cm)	87.8 ± 8.4 (103)	89 ± 11.8 (116)	86.5 ± 9.5 (109)	86.6 ± 10.4 (116)	0.060
HC (cm)	95 ± 6.6 (108)	93.3 ± 9.0 (113)	90.5 ± 7.9 (110)	91.7 ± 8.2 (113)	0.003
SBP (mmHg)	120.9 ± 12.1 (160)	126.5 ± 14.8 (175)	137.1 ± 19.3 (216)	136.2 ± 19.1 (216)	0.000
DBP (mmHg)	82.9 ± 9.2 (112)	82.7 ± 9.5 (110)	85.6 ± 9.9 (110)	84.5 ± 10 (118)	0.019
FBG (mmol/L)	5.8 ± 2.9 (26.9)	7.4 ± 4.3 (24)	7.5 ± 3.2 (18.4)	7.3 ± 3.7 (26.9)	0.000
SUA (mg/dL)	5.6 ± 1.5 (7.9)	5.4 ± 2 (10.1)	5.6 ± 1.6 (10.6)	5.4 ± 1.8 (10.6)	0.304
XO (U/L)	1.6 ± 0.6 (2.3)	3.0 ± 0.4 (3.8)	4.7 ± 0.6 (5.8)	8.4 ± 2.2 (14.6)	0.000
Creatinine (mg/dL)	0.9 ± 0.3 (2.5)	0.9 ± 0.2 (1.3)	0.9 ± 0.3 (2)	0.9 ± 0.3 (2.5)	0.083
TG (mg/dL)	186.7 ± 114.2 (675.2)	179.4 ± 115.4 (583.3)	216.3 ± 131.9(726.6)	199.7 ± 122.8 (812.6)	0.185
TC (mg/dL)	209.2 ± 65.6 (463.5)	234.9 ± 105.6 (562.8)	215.1 ± 89.8 (584.0)	217.7 ± 87.1 (584.0)	0.325
HDL-C (mg/dL)	31.3 ± 9.1 (63.2)	35.4 ± 14 (91)	37.3 ± 17 (112.4)	34.8 ± 13.6 (112.4)	0.063
LDL-C (mg/dL)	141.4 ± 60.2 (324.6)	165.6 ± 97.0 (517.2)	135.3 ± 79.4 (506.5)	144.2 ± 80.3 (517.2)	0.099

Values are presented as mean ± SD (max.). p-values are obtained from one-way ANOVA.

BMI, body mass index; WC, waist circumference; HC, hip circumference; SBP, systolic blood pressure; DBP, diastolic blood pressure; PP, pulse pressure; XO, xanthine oxidase; SUA, serum uric acid; FBG, fasting blood glucose; TG, triglyceride; TC, total cholesterol; HDL-C, high-density lipoprotein cholesterol; LDL-C, low-density lipoprotein cholesterol.

### Association between serum XO and hypertension

Table 4 represents the association between serum XO levels and hypertension. In logistic regression analysis, four models were applied to evaluate the relationship. After adjusting for age and sex in model 1, the odds ratios (ORs) and 95% CI were 1.030 (1.005–1.055), 1.067 (1.041–1.094), and 1.066 (1.040–1.093) respectively in Q2–Q4 compared to Q1. In model 2, after additionally adjusting BMI with model 1, the ORs (95% Cl) were 1.033 (1.007–1.059), 1.075 (1.047–1.103), and 1.073 (1.046–1.102) respectively in Q2–Q4 compared to Q1. In model 3, after adjustment of lipid profile (TG, TC, HDL-C, and LDL-C) with model 2, the ORs (95% Cl) were 1.029 (1.000–1.059), 1.085 (1.054–1.118), and 1.084 (1.052–1.117). In model 4, after adjustment of smoking status with model 3, the ORs (95% Cl) were 1.033 (0.988–1.081), 1.084 (1.037–1.134) and 1.079 (1.031–1.129). In all models, XO showed a positive and independent association with hypertension (p < 0.01).

**Table 4:** Association between XO and hypertension.

	OR (95% CI)				p-values for trend
	Q1	Q2	Q3	Q4	
Model 1	1	1.030 (1.005–1.055)	1.067 (1.041–1.094)	1.066 (1.040–1.093)	< 0.01
Model 2	1	1.033 (1.007–1.059)	1.075 (1.047–1.103)	1.073 (1.046–1.102)	< 0.01
Model 3	1	1.029 (1.000–1.059)	1.085 (1.054–1.118)	1.084 (1.052–1.117)	< 0.01
Model 4	1	1.033 (0.988–1.081)	1.084 (1.037–1.134)	1.079 (1.031–1.129)	< 0.01

Multinomial logistic regression analysis was applied to evaluate the associations between XO and hypertension. Dependent variable was hypertension (yes) and independent variable was XO (U/L). OR, odds ratio; CI, confidence interval; SE, standard error. Model 1: adjusted for age and sex; Model 2: Model 1 plus BMI and SUA; Model 3: Model 2 plus TG, TC, HDL-C, and LDL-C; Model 4: Model 3 plus smoking status.

## Discussion

The present study reports a positive and independent association between serum XO levels and hypertension. This association remained even after adjustment for several confounders such as age, gender, BMI, lipid markers and smoking status. To our knowledge, this study provides the first information on the relationship between serum XO levels and hypertension in Bangladeshi adults.

In our study, the female participants had a higher mean XO level than the male participants. On the other hand, the mean SUA was higher in males than in females. An increased level of SUA has been found in males in several studies^11,14,39,40^. Although there is no established consensus on sex differences in serum XO levels. The differences in the mean level of XO between the sex groups may be related to sex hormones. However, little is known about the relationship between sex hormones and serum XO levels. Visceral fat content adipose tissue is one of the major sources of XOR^41^. Considering that visceral fat mass is generally increased in the postmenopausal stage in women^42^, elderly female participants might have higher serum XO levels. However, further sex-specific mechanistic studies can reveal the exact mechanism for this physiological difference. When study participants were divided by BP levels, the mean level of serum XO was found to be significantly higher in the hypertensive group than in the normotensive group. An increasing trend was observed for SBP and DBP levels across the XO quartiles and XO showed an independent association with hypertension. Our findings are consistent with a recent study that showed also a significant association of plasma XOR with elevated BP in Japanese adults who were not taking antihypertensive or anti-hyperuricemic drugs^24^. This independent association between plasma XOR and hypertension was also found in nondiabetic individuals in another recent study in Japan^25^. However, these two studies could not provide information, on whether taking antihypertensive drugs affects XO levels. In the current study, we also included participants who were receiving antihypertensive medications and still our analysis showed a higher level of serum XO in hypertensive subjects than in normotensive subjects. Furthermore, in our study, some participants were diabetic, however, the presence of diabetes did not show any significant difference in serum XO level with participants who were non-diabetic. Along with the findings of a previous study^24^, our observations suggest that serum XO levels may increase the risk of elevated BP with or without comorbidities, including diabetes.

In the present study, we also observed an increasing trend of serum XO levels with increasing age in the groups. A study stated that the deterioration of the tissues is generally higher in older people^43^. This may be a cause of higher purine metabolism and elevated XO levels in older individuals than in the younger. However, the mechanism for the age-related relationship between serum XO and BP is yet to be explored.

Hyperuricemia is known to predict hypertension development and using an XOR inhibitor has been shown to lower BP in some studies^44,45^. On the other hand, several^46–49^ but not all studies have found no effect from using uricosuric drugs such as benzbromarone on BP. In cardiovascular patients, XO inhibitor has been proven to be effective in reducing the risk of cardiovascular disease^50^. Although in many studies a significant positive association was found between elevated SUA and hypertension, cardiovascular diseases and chronic kidney diseases^13,15,51^, some other studies found both elevated and low levels of SUA to be associated with cardiovascular disease^18,19^ and renal disease^20,21^. A recent umbrella review covering meta-analyses of observational studies, Mendelian analyses and randomized controlled trials, reported that SUA is truly linked only to gout development and nephrolithiasis^22^. Therefore, SUA has been suggested as a mere biomarker other than a true risk factor for cardiovascular and renal disease including hypertension^22^.

The underlying mechanisms between XO and hypertension are not well established yet. There are some possible ways through which elevated XO levels may increase the risk of hypertension. During purine metabolism, XO produces ROS as byproducts^1,4^ which may increase BP via endothelial dysfunction, vascular inflammation, and structural remodeling^5^. In endothelial cells, the redox state is regulated through a balance between NO and ROS-like superoxide anion radical (${\text{O}}_{{2}}^{ \bullet - }$). NO is a potent vasodilator and its diminished levels upon reaction with ${\text{O}}_{{2}}^{ \bullet - }$ cause increased resistance of arterioles, and eventually leads to hypertension development^6,7^. Furthermore, reduced activation of XO may also assist oxidative stress-related injury in kidney and endothelial cells^23^ which leads to elevated BP. However, the exact mechanisms between XO and the risk of hypertension is remained to explore.

The major strength of the present study was the adjustment for several well-known hypertension risk factors in the regression analysis. Moreover, the data and samples were collected following standard procedures and all the biochemical analyses were done in a single laboratory. Our study had also some limitations. First, the cross-sectional nature of this study may not prove the causal relationships between serum XO levels and hypertension. Therefore, a future longitudinal study may provide better insights into the link between XO levels and hypertension. Second, the sample size of this study was relatively small, which does not represent the whole Bangladeshi population. Third, the findings of our study may not be applied to other ethnic groups. Despite several limitations, this study findings may be a worthy reference for future investigations in this area.

## Conclusion

In the present study, a higher mean serum XO level was found in hypertensive individuals and this elevation was higher in females than in males. Serum XO levels were positively correlated with both SBP and DBP. In regression analysis, serum levels of XO showed a positive and independent association with hypertension. Our findings indicate that XO may have an important role in the pathophysiology of elevated blood pressure through generating of ROS. However, more studies are needed to reveal the underlying mechanisms between XO and the risk of hypertension.

## Data Availability

The datasets used and/or analysed during the current study available from the corresponding author on reasonable request.
